# Prediction of Interactions between Viral and Host Proteins Using Supervised Machine Learning Methods

**DOI:** 10.1371/journal.pone.0112034

**Published:** 2014-11-06

**Authors:** Ranjan Kumar Barman, Sudipto Saha, Santasabuj Das

**Affiliations:** 1 Biomedical Informatics Centre, National Institute of Cholera and Enteric Diseases, Kolkata, West Bengal, India; 2 Division of Clinical Medicine, National Institute of Cholera and Enteric Diseases, Kolkata, West Bengal, India; 3 Bioinformatics Centre, Bose Institute, Kolkata, West Bengal, India; International Centre for Genetic Engineering and Biotechnology (ICGEB), India

## Abstract

**Background:**

Viral-host protein-protein interaction plays a vital role in pathogenesis, since it defines viral infection of the host and regulation of the host proteins. Identification of key viral-host protein-protein interactions (PPIs) has great implication for therapeutics.

**Methods:**

In this study, a systematic attempt has been made to predict viral-host PPIs by integrating different features, including domain-domain association, network topology and sequence information using viral-host PPIs from VirusMINT. The three well-known supervised machine learning methods, such as SVM, Naïve Bayes and Random Forest, which are commonly used in the prediction of PPIs, were employed to evaluate the performance measure based on five-fold cross validation techniques.

**Results:**

Out of 44 descriptors, best features were found to be domain-domain association and methionine, serine and valine amino acid composition of viral proteins. In this study, SVM-based method achieved better sensitivity of 67% over Naïve Bayes (37.49%) and Random Forest (55.66%). However the specificity of Naïve Bayes was the highest (99.52%) as compared with SVM (74%) and Random Forest (89.08%). Overall, the SVM and Random Forest achieved accuracy of 71% and 72.41%, respectively. The proposed SVM-based method was evaluated on blind dataset and attained a sensitivity of 64%, specificity of 83%, and accuracy of 74%. In addition, unknown potential targets of hepatitis B virus-human and hepatitis E virus-human PPIs have been predicted through proposed SVM model and validated by gene ontology enrichment analysis. Our proposed model shows that, hepatitis B virus “C protein” binds to membrane docking protein, while “X protein” and “P protein” interacts with cell-killing and metabolic process proteins, respectively.

**Conclusion:**

The proposed method can predict large scale interspecies viral-human PPIs. The nature and function of unknown viral proteins (HBV and HEV), interacting partners of host protein were identified using optimised SVM model.

## Introduction

Viral pathogens affect their eukaryotic host partly by interacting with the proteins of the host cells [1]. Virus-host PPIs are crucial for better understanding of the mechanisms and pathogenesis of infectious diseases [2]. Several computational methods have been proposed to predict protein-protein interactions, but most are designed for intra-species PPIs and only a few for inter-species PPIs. Widely used machine-learning methods for PPIs are SVM, Naïve Bayes and Random forest [3], [4], [5]. Shen et al. used protein sequence information to predict human PPIs by employing SVM with a kernel function and a conjoint triad method, in which the best model predicted with an average accuracy of 83.90% [6]. Guo et al. predicted yeast PPIs with an accuracy of 88.09% using auto covariance (AC) and support vector machines (SVM) [7]. In contrast, Wu et al. predicted yeast PPIs by mining the knowledge of functional associations from the GO-based annotations [8]. Jansen et al. has developed a Bayesian networks approach to predict PPIs in yeast [4], while Lin et al. shows that Random Forest (RF) model may be more effective than Bayesian networks for predicting PPIs [5]. In addition, a number of computational methods are also available in order to predict PPIs based on domain information [9]–[11]. However, relatively few methods have so far been proposed to predict interspecies (specifically host-pathogen) PPIs [3], [12]–[16]. For example, Cui et al. used relative frequency of amino acid triplets of protein sequence to predict the interactions between two types of viruses (hepatitis C virus and human papillomaviruses) and human proteins [3]. Proposed SVM methods of Cui et al. had an accuracy yield over 80%. Dyer et al. also proposed a method to predict physical interactions between human and HIV proteins based on a number of features, such as domain profiles, protein sequence k-mers and properties of human proteins in a human PPI network [12]. At a precision value of 70%, their method achieved recall (sensitivity) values of 40%.

In this paper, we have made an attempt to predict viral-host (inter-species) PPIs based on three well-known supervised machine-learning methods, namely SVM, Naïve Bayes and Random Forest using significantly diverse biological information like protein sequence, domain-domain associations, disorder regions, degree and amino-acids composition of viral and human proteins. The viral-host PPIs dataset were obtained from VirusMINT, a viral protein interaction database [17]. We have shown that only four features can able to predict viral-host PPIs with high degree of accuracy, which is comparable to the existing prediction models for viral-host PPIs. Furthermore we have shown that the viral protein amino acids composition (methionine, serine and valine) plays an important role in viral-host PPIs. An attempt was made to predict unknown PPIs between hepatitis B virus (HBV)-human proteins and hepatitis E virus (HEV)-human proteins using our proposed SVM optimal model. Predicted significant protein pairs were grouped using hierarchical clustering analysis (HCA) and validated using gene ontology enrichment analysis. Overall, the proposed support vector machines (SVM)-based machine learning technique was able to predict unknown viral-host protein interaction pairs with reasonable accuracy, which may be subjected to experimental validation.

## Materials and Methods

### 2.1 Datasets

#### 2.1.1 Data preparation

The dataset used were obtained from “VirusMINT: a viral protein interaction database” (ftp://mint.bio.uniroma2.it/pub/virusmint/MITAB/current/2012-10-26-mint-viruses-binary.mitab26.txt) [17]. VirusMINT database emphasises on interaction between human and some of the medically significant viruses: human immunodeficiency virus 1 (HIV-1), simian virus 40 (SV40), hepatitis B virus (HBV), hepatitis C virus (HCV), papilloma virus. Unique and positive 1,146 viral-host PPIs were derived from initial 2,707 interactions, after eliminating 1,224 repetitive interactions (Vprot A-Hprot B and Hprot B-VprotA) and 337 interacting protein pairs not having any “InterPro” domain hit. Out of these, 1,035 interactions were found between viral and human proteins and 111 interactions between viral proteins and proteins of others species including mouse, rat, dog and bovine. Furthermore, non-redundant interaction analysis based on the homologous viral proteins present in training and testing sets showed that 0.77% of the viral-human PPIs were redundant (shown in Table S1). We used cd-hit-2d webserver (http://weizhong-lab.ucsd.edu/cdhit_suite/cgi-bin/index.cgi?cmd=cd-hit-2d) at 85% sequence identity level to find the homologous proteins present in the training and testing sets [18]. Since, large numbers of viral-human PPIs were distinct (99.23%) and there were only few (1,035) viral-human PPIs in the initial set, we considered all 1,035 positive interactions between the viral and human proteins as training and testing datasets in our 5-fold cross-validation study (Table S2).

#### 2.1.2 Negative training and testing dataset

Ben-Hur et al. [19] proposed that in the case of predicting protein-protein interactions, a simple uniform random choice of non-interacting protein pairs yield an unbiased estimate of the true distribution. In absence of experimentally proven non-interacting protein pairs, which are considered as an ideal negative dataset, we choose random 1,035 viral-human protein pairs that were not found in the positive training and testing datasets in our study as the negative dataset. In order to avoid prediction bias, we generated a negative dataset with the same number of viral-human PPIs (positive:negative = 1:1) as the positive dataset (Table S3).

#### 2.1.3 Blind dataset

111 positive interactions between viral and non-human species proteins, which were not used in 5-fold cross-validation, were considered as a blind dataset to avoid overfitting problem in building our optimal model for predictions (Table S4). Non-redundant interaction analysis, based on the homologous proteins present in the training and blind sets showed that 8.11% interactions between the viral and non-human species proteins were redundant (shown in Table S1). Therefore we removed 9 redundant interactions between the viral and non-human species proteins from the blind dataset. Like the negative training and test dataset, 102 negative viral and non-human species protein pairs were also generated (Table S5).

#### 2.1.4 Independent dataset

In order to predict unknown viral and human PPIs, we focused on some of the medically significant viruses, such as hepatitis B and hepatitis E. Instead of taking all the proteins of hepatitis B, we concentrated on the proteins of hepatitis B virus genotype C that is prevalent in the eastern India [20]. Thus, reviewed 4 hepatitis B virus proteins (genotype C) with InterPro domain hits were obtained from Swiss-Prot [21]. Begum et al. observed that hepatitis E virus genotype 4 ‘e’ is prevalent in the Northern India [22], while Caron et al. found that genotype 1 of hepatitis E virus is most prevalent in the Asian countries [23]. Hence, reviewed 3 hepatitis E virus proteins (genotype 4 ‘e’) and 3 hepatitis E virus proteins (genotype 1) with InterPro domain hits were retrieved from Swiss-Prot. Reviewed 17,615 human proteins with InterPro domain hits were also retrieved from Swiss-Prot.

### 2.2 Machine Learning Techniques (MLT)

We focused on three well-known supervised machine learning methods, such as SVM, Naïve Bayes and Random Forest that were used for predicting PPIs [3], [4], [5].

#### 2.2.1 SVM

Support Vector Machines (SVM)-based method is defined over a vector space where the problem is to find a decision surface that maximizes the margin between data points in the two classes. We have used SVM^light^ tool provided by T. Joachims [24], which allows users to select various parameters and various kernel functions like linear, polynomial, radial basis function (RBF), sigmoid to find optimal parameters for each task.

#### 2.2.2 Naïve Bayes

Naïve Bayes model computes subsequent probabilities for a given hypothesis (present/absence) assuming that the features that describe data instances are conditionally independent. Its performance is comparable to other supervised learning methods. We used Waikato Environment for Knowledge Analysis (WEKA) machine learning tool box to perform Naïve Bayes classification [25]. Since WEKA does not allow us to select different parameters set for Naïve Bayes classification, we used the default parameters set.

#### 2.2.3 Random Forest

Random Forest (RF) classifier “grows” several Decision Trees (DTs) simultaneously where each node uses a random subset of the features. Each tree in the Random Forest classifies the new object, and “votes” for that class. The forest elects the classification based on majority vote (over all the trees in the forest). We obtained Random Forest (RF) classifier from the WEKA machine learning tool box. Optimal parameters were used for evaluation of the method.

### 2.3 Feature Vectors

We focused on forty-four features of protein pairs to produce feature vectors (Table S6). First, occurrence frequency of viral-host domain-domain association was used since domain-domain association plays an important role in protein-protein interactions [11]. Second, common domains observed in virus and host proteins were chosen and represented as binary format [0,1] (absence and presence of common domain observed in virus and host proteins in a particular protein pair represented by 0 and 1, respectively). Third, maximum degree of viral or human protein for a given viral-human protein pair was selected. Degrees of human proteins were collected from APID2NET (a Cytoscape plugin) and viral protein degrees were collected from viral-host PPIs. APID2NET provided us all possible PPIs from BIND, BioGrid, DIP, HPRD, IntAct and MINT databases [26]. Fourth, average percentages of disorder regions of protein pairs were selected, because intrinsically disordered proteins were found to be implicated in numerous cellular pro­cesses including signal transduction, transcriptional regulation and PPIs. We used “ESpritz: accurate and fast prediction of protein disorder” to gather percentage of disorder regions of proteins [27]. Finally, amino acid compositions of viral and host proteins were selected as a fifth to twenty-fourth and twenty-fifth to forty-fourth features of our proposed feature vectors. Since, Roy et al. proposed that amino acid composition (AAC) monomers feature is crucial for predicting PPIs [28].

### 2.4 Infer domain-domain associations

We inferred viral and host domain-domain associations from interacting protein pairs. Our goal was to find the frequency of a certain viral and host domain-domain association present in protein pairs. We collected all protein related “InterPro” domains from Protein Knowledgebase, UniProtKB (http://www.uniprot.org/) [21]. After retrieving the “InterPro” domain information, we computed viral domain-human domain association matrix (rows and columns represented host and viral domain names, respectively), using similar approach proposed by Sprinzak et al. [29]. The range of domain-domain association varies between 30 and 1, where 0 represents no association. We tried with two domain-domain association scores, such as Maximum Domain-Domain Association Score (MDDAS) and Average Domain-Domain Association Score (ADDAS). The MDDAS and ADDAS were calculated using following equations:
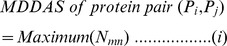


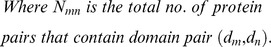


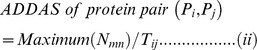


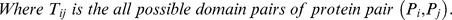



### 2.5 Amino acid composition

Amino acid composition is the percentage of each amino acid present in a protein. Percentage of all twenty natural amino acids was calculated using the following equations:
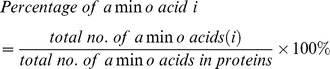


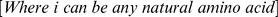



### 2.6 Feature selection

The feature selection was performed by regression for categorical data method with beta coefficient >0.00 and p-value<0.05 for selection of best features using SPSS statistical analysis software, version 20 (SPSS, Chicago, IL, USA). The beta coefficient value is a measure of how strongly each “predictor variable” influences the “criterion variable”. The higher beta coefficient value implies greater impact of the “predictor variable” on the “criterion variable”.

### 2.7 5-fold cross-validation

We used 5-fold cross-validation to estimate performance of all methods. In 5-fold cross-validation, the dataset has been partitioned into 5 equally (or nearly equally) sized segments or folds. Consequently, 5 times of training and testing were performed such that each time a different fold of the data is held-out for testing while the remaining four folds are used for training. The overall performance of a method was calculated using average performance over five folds.

### 2.8 Performance measures

#### 2.8.1 Threshold Dependent

Sensitivity (also referred to as recall), specificity, accuracy, PPV (Positive Prediction Value, also referred to as precision), Matthew’s correlation coefficient (MCC) and F1 score were computed on 5-fold cross validation step. All the performance measures were based on a balanced dataset of 1∶1 positive vs. negative examples. Sensitivity, specificity, accuracy, PPV, MCC and F1 score were calculated by the following equations:
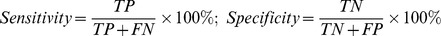


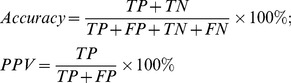


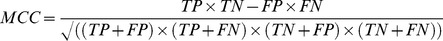


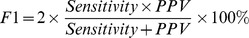




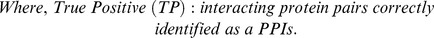
 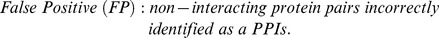 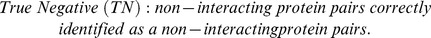 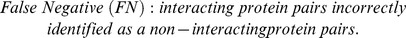


#### 2.8.2 Threshold independent

From Receiver Operating Characteristic (ROC) plot**,** area under ROC curve was computed on 5-fold cross validation step.

### 2.9 Hierarchical clustering analysis (HCA)

Hierarchical clustering analysis was done using TIBCO Spotfire software [30]. The input matrix was viral-host SVM prediction scores obtained from the best optimized model. Following parameters were used for HCA: complete linkage clustering method, cosine correlation distance measure, average value ordering weight, scale between 0 and 1 normalization and empty value replace by 0 for both (row and column) dendrogram.

### 2.10 GO Enrichment analysis

The Database for Annotation, Visualization and Integrated Discovery (DAVID) web server was used to identify significantly enriched gene ontology (GO) annotation terms in predicted interacting human protein partners of hepatitis B and E viruses [31]. We consider only GO biological process annotation terms of level greater than 2 with significant false discovery rate (FDR) value<0.05.

## Results and Discussion

### 3.1 Selection of optimal features

We started with 44 features of a specific viral-host protein pair and tried with different subsets of features in order to achieve maximum accuracy with nearly equal sensitivity and specificity of our proposed method (Table S6, S7). Interestingly, we observed that four features with beta coefficient>0.00 and p-value<0.05 showed reasonably decent accuracy of 71%, sensitivity of 67% and specificity of 74% in proposed SVM method (shown in Table 1). As shown in Table 2, selected four features achieved slightly higher accuracy than all the forty-four features used together. Although disordered regions play a significant role in protein-protein interactions, it was not selected as the best feature based on our feature selection with regression (beta coefficient and p-value). Methionine residue interacts with aromatic residues and plays a specific role in stabilization of protein structure and may be associated with number of mutation and age related diseases [32]. Serine residues are crucial for serine/threonine protein phosphatises and control many cell functions [33], while valine residue was shown to play a vital role in modulating syncytium formation during infection [34].

**Table 1:** List of best 4 features selected based on categorical regression method.

Features	Beta	Bootstrap (1000)Estimate of Std. Error	df	F	Sig.(P_Value_)
Average domain-domainassociation score	0.511	0.016	1.000	982.607	0.000
Virus Methionine	0.070	0.021	1.000	10.911	0.001
Virus Serine	0.106	0.021	1.000	25.838	0.000
Virus Valine	0.094	0.023	1.000	16.829	0.000

**Table 2:** Comparison of performance between selected best 4 features vs all 44 features.

Method	All Features			Selected Features		
	Accuracy(%)	Area underROC curve	F1 Score(%)	Accuracy(%)	Area underROC curve	F1Score (%)
Naïve Bayes	67.48	0.66	56.72	68.50	0.71	54.35
SVM	**68.00**	**0.72**	**65.04**	**71.00**	**0.73**	**69.41**
Random Forest	71.69	0.77	67.13	72.41	0.76	66.39

### 3.2 Performance of SVM, NB and RF using 5-fold cross validation

In order to achieve optimal sensitivity, specificity and accuracy, we tried different kernels and parameters using SVM. The linear and polynomial kernel function showed high specificity, but low sensitivity, whereas the sigmoid kernel function exhibited poor sensitivity (Table S8). In contrast, the radial basis function (RBF) showed reasonable sensitivity of 67%, specificity of 74% and accuracy of 71% as shown in Table 3. We tried with different parameters in WEKA for Random Forest (shown in Table S9). SVM had nearly equal sensitivity (67%) and specificity (74%), whereas Naïve Bayes and Random Forest showed lower sensitivity (37.49% for NB, 55.66% for RF), but higher specificity (99.52% for NB, 89.08% for RF) (Table 4). As shown in Table 4, Random Forest perform better in terms of accuracy, MCC and area under ROC curve, whereas SVM perform better in terms of sensitivity and F1 score. We are more concerned about the recall and precision, since they are directly proportional to the true positives. As shown in Table 4, recall score of SVM (67%) is better than RF (55.66%), while precision score of RF (82.26%) is better than SVM (72%). Therefore we computed the F1 score. F1 score of SVM and RF shows that, SVM (69.41) performs slightly better than RF (66.39). Therefore, we used the best SVM model for further study.

**Table 3:** SVM based performance on testing dataset (5-fold cross-validation) using parameters t = 2 (RBF kernel), and g = 1, c = 0.1, j = 2.

Threshold	Sensitivity (%)	Specificity (%)	Accuracy (%)	PPV (%)	MCC
0.8	37	91	64	76	0.32
0.7	46	89	67	78	0.39
0.6	52	85	68	76	0.40
0.5	59	80	69	75	0.42
**0.4**	**67**	**74**	**71**	**72**	**0.44**
0.3	69	70	70	69	0.42
0.2	73	65	69	68	0.41
0.1	76	59	68	65	0.38
0	80	51	66	62	0.35
-0.1	81	46	64	60	0.32
-0.2	83	40	62	58	0.28
-0.3	85	36	60	57	0.26
-0.4	87	29	58	55	0.23
-0.5	89	25	57	54	0.20
-0.6	89	20	54	53	0.15
-0.7	91	16	53	52	0.12
-0.8	91	11	51	51	0.08

**Table 4:** Comparison of performance measures among Naïve Bayes, SVM and Random Forest methods on testing dataset using 5-fold cross-validation technique in our study.

Methods	Sensitivity (%)	Specificity (%)	Accuracy (%)	PPV (%)	MCC	Area under ROC curve	F1 Score (%)
Naïve Bayes	37.49	99.52	68.50	98.80	0.47	0.71	54.35
SVM^light^	**67.00**	**74.00**	**71.00**	**72.00**	**0.44**	**0.73**	**69.41**
Random Forest	55.66	89.08	72.41	82.26	0.48	0.76	66.39

### 3.3 Assessment on blind dataset using SVM based method

In order to avoid bias in the performance of our proposed model, we tested it on blind dataset, not used in training or testing. Consequently, 204 protein pair between viral proteins and non-human species (mouse, rat, dog, bovine etc.) was considered as a blind dataset. We used the same parameters and cut-off (threshold) for each approach. As shown in Table 3, threshold value of 0.4 generated reasonable accuracy on the test dataset using 5-fold cross-validation technique in our study. At this threshold value, sensitivity of 64%, specificity of 83%, and accuracy of 74% was achieved on the blind dataset.

### 3.4 Comparison with other predictions methods for virus-host PPIs

Dyer et al. developed a method to predict HIV-human PPIs using SVM classifier with linear kernel on different combinations of protein features, including domain profiles, protein sequence k-mers and properties of human proteins in a human PPI network [12]. They predicted PPIs with a precision of 70% and a recall (also referred to as sensitivity) value greater of 40% using a combination of protein sequence four-mers, protein domains and PPI network information with 1∶25 ratio of positive example (PE) to negative example. We obtained only 332 positive interactions instead of 1028 interactions reported by Dyer et al. between human and HIV proteins [12]. As shown in Table 5, our proposed method achieved the sensitivity of 87% whereas Dyer et al. achieved 40%.

**Table 5:** Comparison of proposed method with other viral-host PPIs prediction methods.

Performance Mesaure	Dyer et al. Dataset*		Performance Mesaure	Cui et al. Dataset*		
	Dyer et al. [12]	Proposed SVM Model		Shen et al. [6]	Proposed SVM Model	Cui et al. [3]
Sensitivity (%)	40.00	87.05	Accuracy (%)	78.00	80.00	82.00

*Partial dataset.

Cui et al. worked on a similar problem of HCV-human and HPV-human PPIs using an SVM model with RBF kernel and relative frequency of amino acid triplets of a protein sequence. They have used 11 HCV (lead to 695 interactions) and 9 HPV proteins (lead to 252 interactions) [3]. From the available datasets in the supplementary tables, we can extract 1 HCV protein (leads to 10 positive and 9 negative interactions) and 1 HPV protein (leads to 9 positive and 7 negative interactions) from Swiss-Prot. Our proposed method achieved accuracy of 80% on this sparsely available dataset (shown in Table 5), whereas Shen et al. and Cui et al. achieved accuracy of 78% and 82%, respectively [3], [6].

### 3.5 Prediction of unknown HBV-human and HEV-human PPIs

**Figure 1:**
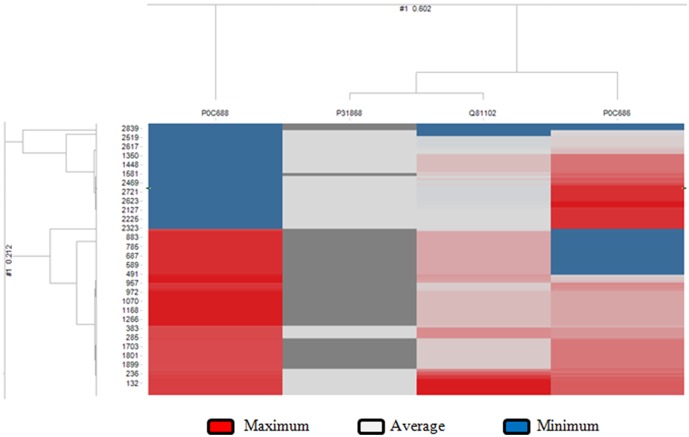
Hierarchical clustering of highly predicted SVM score of HBV-human protein pairs. Hierarchical clustering analysis was done using TIBCO Spotfire software with complete linkage clustering method, cosine correlation distance measure, average value ordering weight, scale between 0 and 1 normalization and empty value replace by 0 for both (row and column) dendrogram. The high, average and low SVM predicted scores are marked in red, white and blue, respectively.

Hepatitis B virus and hepatitis E virus proteins were used in order to predict unknown viral-human PPIs. All possible combinations of hepatitis B virus and human protein pairs (17615 * 4) were predicted by our proposed model (Table S10). The predicted SVM score greater than 0.58 of hepatitis B virus-human protein pairs (n = 8411) was used for HCA. As shown in Figure 1, P0C688 (gene name P) was far apart from the other three hepatitis B viral proteins, out of which P31868 (gene name S) and Q81102 (gene name C) are closely associated. Similarly, all combinations of hepatitis E virus and human protein pairs (17615 * 6) were predicted by the proposed model (Table S11). The highly predicted SVM score of hepatitis E virus and human protein pairs were used for HCA. In HEV-host interaction pairs (n = 20,375) clustering analysis, Q9IVZ7 (gene name ORF3 and genotype 4) was far apart from the other five hepatitis E viral proteins, out of which Q9IVZ8 (gene name ORF2, genotype 4), Q9IVZ9 (gene name ORF1, genotype 4), P33424 (gene name ORF1, genotype 1) and P33426 (gene name ORF2, genotype 1) were closely associated (Figure S1).

**Figure 2:**
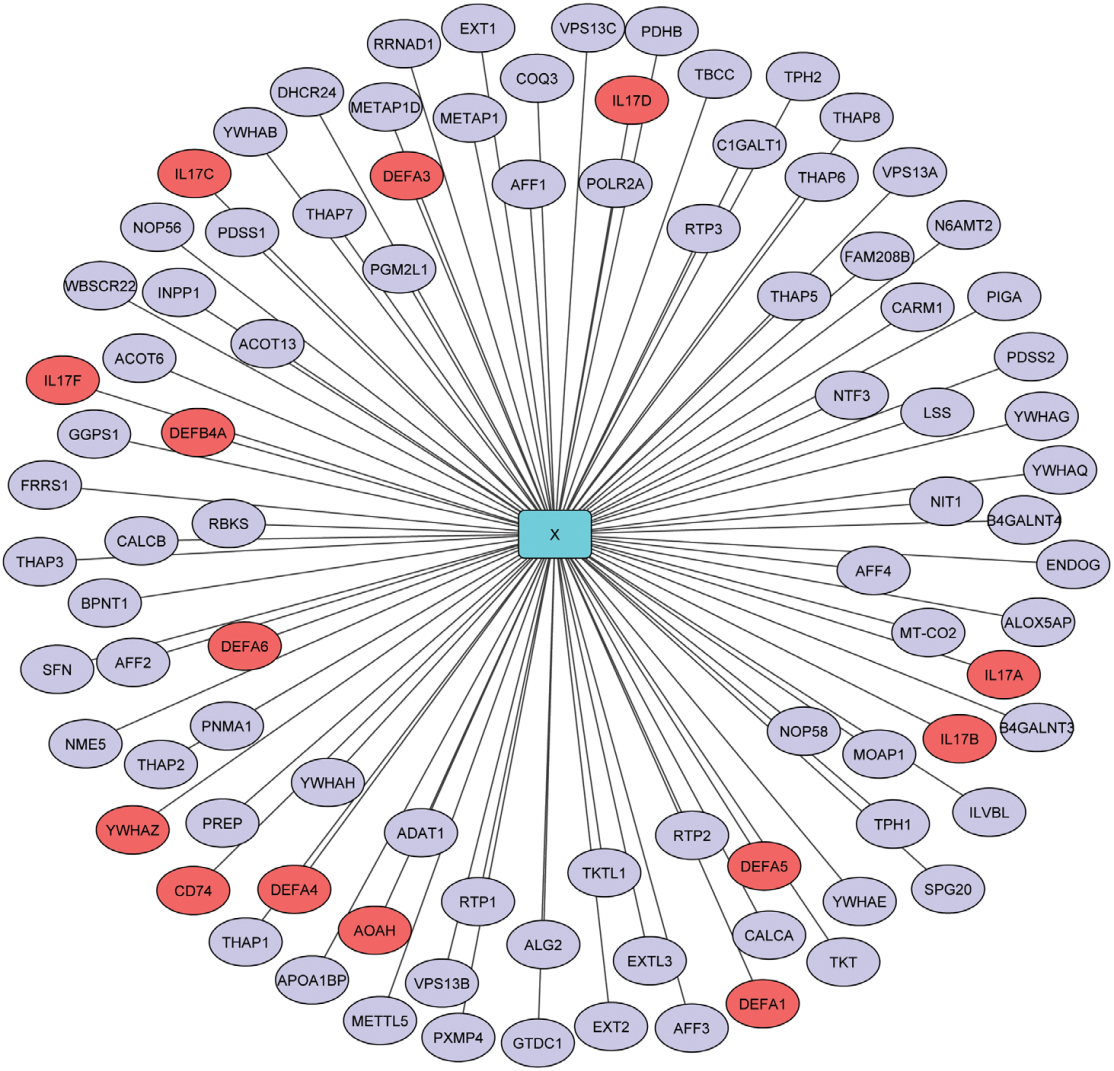
A network of HBX-human protein interactions predicted by our proposed method. The network visualized by Cytoscape 3.0.2 [35]. The HBX protein is represented by cyan node. The significant gene ontology enriched human proteins are representing by salmon node, whereas other human proteins are representing by slate grey node.

Finally, the human proteins present in high confidence (red area) of hierarchical clustering analysis (Shown in Figure 1 and Figure S1) were used for further gene ontology enrichment analysis. The analysis on interacting human protein partners of hepatitis B virus (Shown in Figure 2 and Figure S2, S3) showed probable functions of viral “X protein” (UniProtKBId: P0C686), “C protein” (UniProtKBId: Q81102) and “P protein” (UniProtKBId: P0C688) (shown in Table 6 and full data on Table S12-S14). As shown in Table 6, HBV “C proteins” probably plays a significant role in membrane docking, while “X protein” and “P protein” function in cell killing and modulating metabolic processes of host proteins, respectively.

**Table 6:** The Gene Ontology Biological Process enrichment analysis on interacting human protein partners of HBV proteins using DAVID server.

HepatitisB virusprotein	GOterm∼BiologicalProcess	Human protein
C	GO:0022406∼membrane docking	SCFD1, SCFD2, VPS45, STXBP1, STXBP2, STXBP3
	GO:0006835∼dicarboxylic acid transport	SLC1A4, SLC1A5, SLC1A2, SLC1A3,SLC1A1
	GO:0006865∼amino acid transport	SLC1A4, CPT1B, SLC1A5, SLC1A2,CPT2, SLC1A3, XK, SLC1A1
X	GO:0001906∼cell killing	DEFA6, DEFA5, DEFA4, DEFA3, DEFA1
	GO:0009620∼response to fungus	DEFA6, DEFA5, DEFA4, DEFA3, DEFA1
	GO:0006952∼defense response	YWHAZ, DEFB4A, CD74, IL17C,IL17D, IL17A, IL17B, DEFA6, AOAH, DEFA5, DEFA4, IL17F, DEFA3, DEFA1
P	GO:0051186∼cofactor metabolic process	NAMPT, ACO2, HMGCR, ACO1,IREB2, GIF, PNP, SOD2, SDHA,GSS, MTHFS, PGLS, PANK2, PANK3, FXN, PANK1, NARFL, CTNS, NAPRT1, FH
	GO:0006732∼coenzyme metabolic process	NAMPT, ACO2, HMGCR, ACO1, PNP, SOD2, SDHA, GSS, MTHFS, PGLS, PANK2, PANK3, PANK1, CTNS, NAPRT1, FH

Significant biological process annotation terms were filtered by FDR (false discovery rate) <0.05.

Similar study as hepatitis B virus proteins was also done with hepatitis E virus proteins (Shown in Figure S4-S9), where ORF1 (genotype 1) is probably involved in many biological processes including regulation of cytoskeleton organization, nitrogen compound biosynthetic process and translation (Shown in Table S15 and full data on Table S16-S21).

## Conclusion

Here, we proposed three supervised machine learning-based techniques for predicting viral-host (across species) PPIs by incorporating potential biological information of protein pairs including domain-domain associations score, degree, percentage of disorder regions and amino acid compositions. Initially, we started with 44 features and predicted four best features, which were domain-domain association and methionine, serine and valine amino acid composition of viral proteins using categorical regression model (beta coefficient>0.00 and p-values<0.05). There are biological interpretations of these residues of viral proteins for their importance in viral-host PPIs. For example, methionine, serine and valine may be involved in stabilization of the protein structure, serine/threonine protein phosphatases and modulating syncytium formation during infection, respectively. It was observed that Random Forest perform better in terms of accuracy, MCC and area under ROC curve, while the proposed SVM method performs better in terms of sensitivity and F1 score. Performance of the proposed SVM method was evaluated on the blind dataset of 204 viral-host protein pairs (102 positive and 102 negative viral-host protein pairs), which achieved a sensitivity of 64%, specificity of 83%, and accuracy of 74%. In addition, unknown HBV-human and HEV-human PPIs were predicted using optimised SVM model and were grouped by HCA and further validated by GO enrichment analysis. Hepatitis B virus interacting human proteins show distinct GO biological process terms; for example, “X-protein” probably interferes with cell defence mechanism, whereas “P-protein” binds to metabolic pathways. The predicted viral-human PPIs give us hint about the possible role of viral proteins in the pathogenesis process.

## Supporting Information

Figure S1 **Hierarchical clustering of highly predicted SVM score of HEV-human protein pairs.** Hierarchical clustering analysis was done using TIBCO Spotfire software with complete linkage clustering method, cosine correlation distance measure, average value ordering weight, scale between 0 and 1 normalization and empty value replace by 0 for both (row and column) dendrogram. The high, average and low SVM predicted scores are marked in red, white and blue, respectively.
(PDF)

Figure S2 **A network of HBC-human protein interactions predicted by our proposed method.** The network visualized by Cytoscape 3.0.2 [35]. The HBC protein is representing by cyan node. The significant gene ontology enriched human proteins are representing by salmon node, whereas other human proteins are representing by slate grey node.
(PDF)

Figure S3 **A network of HBP-human protein interactions predicted by our proposed method.** The network visualized by Cytoscape 3.0.2 [35]. The HBP protein is representing by cyan node. The significant gene ontology enriched human proteins are representing by salmon node, whereas other human proteins are representing by slate grey node.
(PDF)

Figure S4 **A network of HEORF1 (Genotype 1)-human protein interactions predicted by our proposed method.** The network visualized by Cytoscape 3.0.2 [35]. The HEORF1 (Genotype 1) protein is representing by cyan node. The significant gene ontology enriched human proteins are representing by salmon node whereas other human proteins are representing by slate grey node.
(PDF)

Figure S5 **A network of HEORF2 (Genotype 1)-human protein interactions predicted by our proposed method.** The network visualized by Cytoscape 3.0.2 [35]. The HEORF2 (Genotype 1) protein is representing by cyan node. The significant gene ontology enriched human proteins are representing by salmon node whereas other human proteins are representing by slate grey node.
(PDF)

Figure S6 **A network of HEORF3 (Genotype 1)-human protein interactions predicted by our proposed method.** The network visualized by Cytoscape 3.0.2 [35]. The HEORF3 (Genotype 1) protein is representing by cyan node. The significant gene ontology enriched human proteins are representing by salmon node whereas other human proteins are representing by slate grey node.
(PDF)

Figure S7 **A network of HEORF1 (Genotype 4)-human protein interactions predicted by our proposed method.** The network visualized by Cytoscape 3.0.2 [35]. The HEORF1 (Genotype 4) protein is representing by cyan node. The significant gene ontology enriched human proteins are representing by salmon node whereas other human proteins are representing by slate grey node.
(PDF)

Figure S8 **A network of HEORF2 (Genotype 4)-human protein interactions predicted by our proposed method.** The network visualized by Cytoscape 3.0.2 [35]. The HEORF2 (Genotype 4) protein is representing by cyan node. The significant gene ontology enriched human proteins are representing by salmon node whereas other human proteins are representing by slate grey node.
(PDF)

Figure S9 **A network of HEORF2 (Genotype 4)-human protein interactions predicted by our proposed method.** The network visualized by Cytoscape 3.0.2 [35]. The HEORF2 (Genotype 4) protein is representing by cyan node. The significant gene ontology enriched human proteins are representing by salmon node whereas other human proteins are representing by slate grey node.
(PDF)

Table S1 **Statistic of homologous protein present in the training and testing sets as well as from the blind datasets.**
(XLSX)

Table S2 **Positive interactions dataset used in this study to build optimal model for prediction.** The positive interactions dataset used in the study were obtained from VirusMINT.
(XLSX)

Table S3 **Negative interactions dataset used in this study to build optimal model for prediction.** The negative interactions dataset used in the study were chosen using random protein pairs which are not found in interacting protein pairs.
(XLSX)

Table S4 **Positive interactions dataset used in this study as a positive blind dataset.** The positive blind dataset used in the study were obtained from VirusMINT.
(XLSX)

Table S5 **Negative interactions dataset used in this study as a negative blind dataset.** The negative blind dataset used in the study were chosen using random protein pairs which are not found in interacting protein pairs (positive blind dataset).
(XLSX)

Table S6 **All 44 input features.**
(XLSX)

Table S7 **SVM performance measures based on different subsets of features.** Optimal parameters were used for respective subset of features.
(XLSX)

Table S8 **Several SVM kernel-wise performance measures (sensitivity and specificity) on different models.** Optimal parameters and threshold were used for respective kernel. In Model 1, 1^st^, 2^nd^, 3^rd^ and 4^th^ folds were used for training and 5^th^ fold was kept for testing. In Model 2, 1^st^, 2^nd^, 3^rd^ and 5^th^ folds were used for training and 4^th^ fold was left out for testing. In Model 3, 1^st^, 2^nd^, 4^th^, 5^th^ folds were used for training and 3^rd^ fold for testing. In Model 4, 1^st^, 3^rd^, 4^th^, 5^th^ folds were used for training and 2^nd^ fold was used for testing. In Model 5, 2^nd^, 3^rd^, 4^th^, 5^th^ folds were used for training and 1^st^ fold was kept aside for testing.
(XLSX)

Table S9 **Different parameters used in Random Forest using WEKA.**
(XLSX)

Table S10 **Predicted scores of HBV-human protein-protein association by proposed optimal model.**
(XLSX)

Table S11 **HEV-human protein-protein association predicted scores by proposed optimal model.**
(XLSX)

Table S12 **GO enrichment analysis on interacting human protein partners of HBV X proteins using DAVID server.** Significant biological process terms were chosen by P_Value_<0.05.
(XLSX)

Table S13 **GO enrichment analysis on interacting human protein partners of HBV C proteins using DAVID server.** Significant biological process terms were chosen by P_Value_<0.05.
(XLSX)

Table S14 **GO enrichment analysis on interacting human protein partners of HBV P proteins using DAVID server.** Significant biological process terms were chosen by P_Value_<0.05.
(XLSX)

Table S15 **GO enrichment analysis on interacting human protein partners of HEV proteins using DAVID server.** Significant biological process annotation terms were filter by FDR (false discovery rate)<0.05.
(XLSX)

Table S16 **GO enrichment analysis on interacting human protein partners of HEV ORF1 (Genotype 1) proteins using DAVID server.** Significant biological process terms were chosen by P_Value_<0.05.
(XLSX)

Table S17 **GO enrichment analysis on interacting human protein partners of HEV ORF2 (Genotype 1) proteins using DAVID server.** Significant biological process terms were chosen by P_Value_<0.05.
(XLSX)

Table S18 **GO enrichment analysis on interacting human protein partners of HEV ORF3 (Genotype 1) proteins using DAVID server.** Significant biological process terms were chosen by P_Value_<0.05.
(XLSX)

Table S19 **GO enrichment analysis on interacting human protein partners of HEV ORF1 (Genotype 4) proteins using DAVID server.** Significant biological process terms were chosen by P_Value_<0.05.
(XLSX)

Table S20 **GO enrichment analysis on interacting human protein partners of HEV ORF2 (Genotype 4) proteins using DAVID server.** Significant biological process terms were chosen by P_Value_<0.05.
(XLSX)

Table S21 **GO enrichment analysis on interacting human protein partners of HEV ORF3 (Genotype 4) proteins using DAVID server.** Significant biological process terms were chosen by P_Value_<0.05.
(XLSX)
