# Supplementary figures and images for: Prediction of Interactions between Viral and Host Proteins Using Supervised Machine Learning Methods
Source: PLoS One. 2014 Nov 6;9(11):e112034. doi: 10.1371/journal.pone.0112034 (PMC4223108; doi:10.1371/journal.pone.0112034)

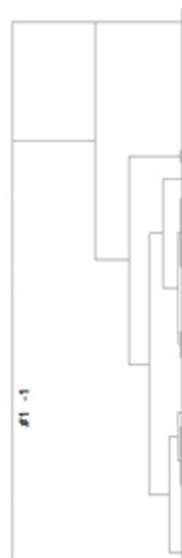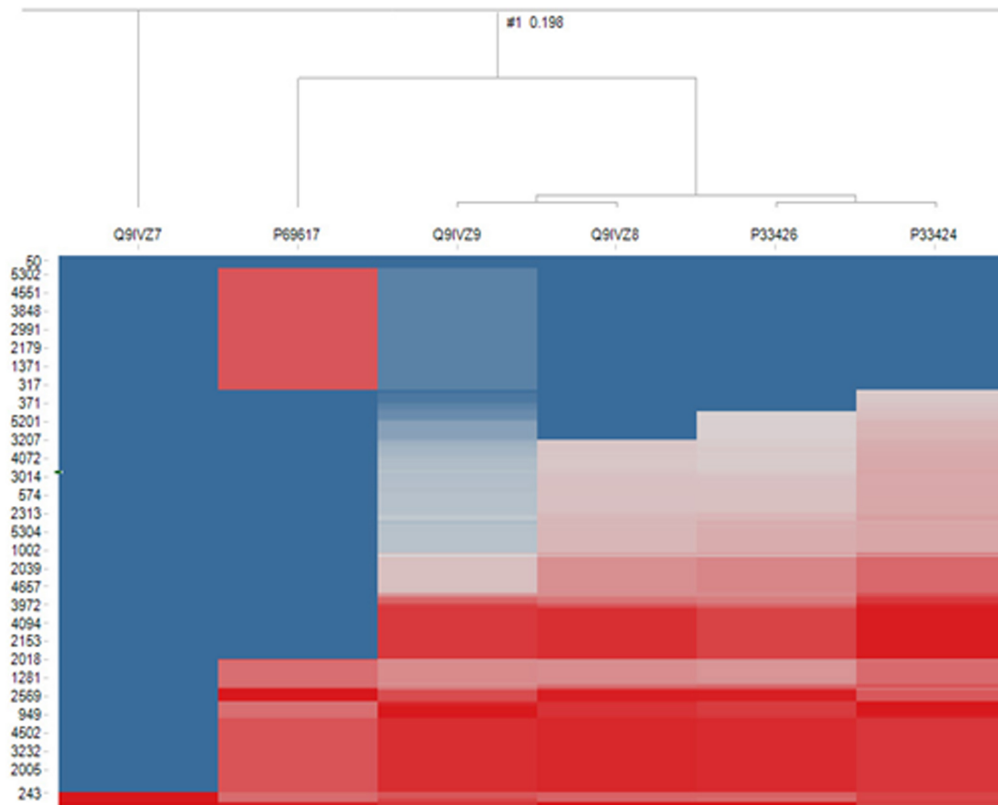

Maximum

Average

Minimum

Supplement: Figure S1 — Hierarchical clustering of highly predicted SVM score of HEV-human protein pairs. Hierarchical clustering analysis was done using TIBCO Spotfire software with complete linkage clustering method, cosine correlation distance measure, average value ordering weight, scale between 0 and 1 normalization and empty value replace by 0 for both (row and column) dendrogram. The high, average and low SVM predicted scores are marked in red, white and blue, respectively. (PDF) [file pone.0112034.s001.pdf]

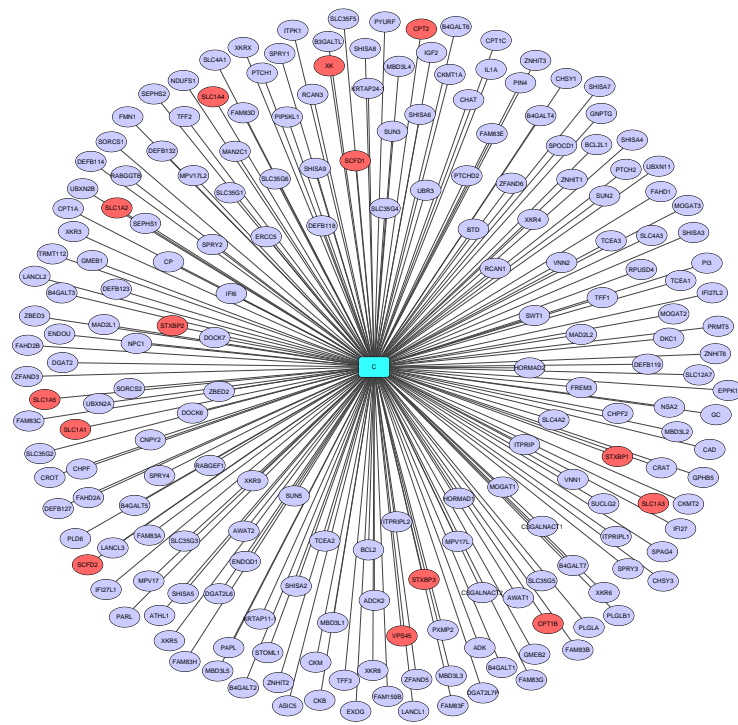

Supplement: Figure S2 — A network of HBC-human protein interactions predicted by our proposed method. The network visualized by Cytoscape 3.0.2 [35]. The HBC protein is representing by cyan node. The significant gene ontology enriched human proteins are representing by salmon node, whereas other human proteins are representing by slate grey node. (PDF) [file pone.0112034.s002.pdf]

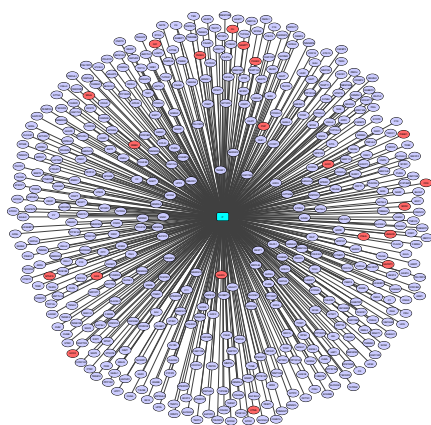

Supplement: Figure S3 — A network of HBP-human protein interactions predicted by our proposed method. The network visualized by Cytoscape 3.0.2 [35]. The HBP protein is representing by cyan node. The significant gene ontology enriched human proteins are representing by salmon node, whereas other human proteins are representing by slate grey node. (PDF) [file pone.0112034.s003.pdf]

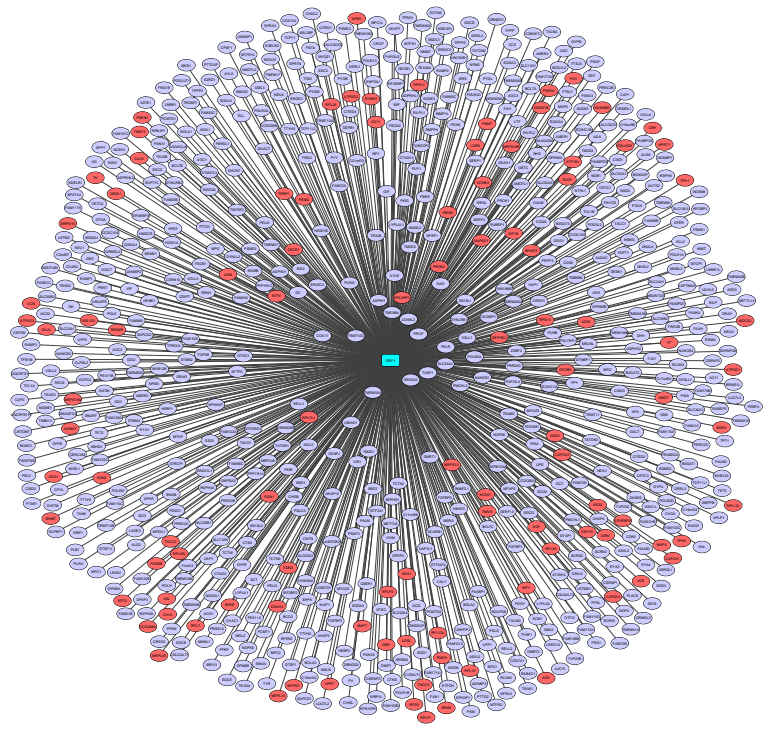

Supplement: Figure S4 — A network of HEORF1 (Genotype 1)-human protein interactions predicted by our proposed method. The network visualized by Cytoscape 3.0.2 [35]. The HEORF1 (Genotype 1) protein is representing by cyan node. The significant gene ontology enriched human proteins are representing by salmon node whereas other human proteins are representing by slate grey node. (PDF) [file pone.0112034.s004.pdf]

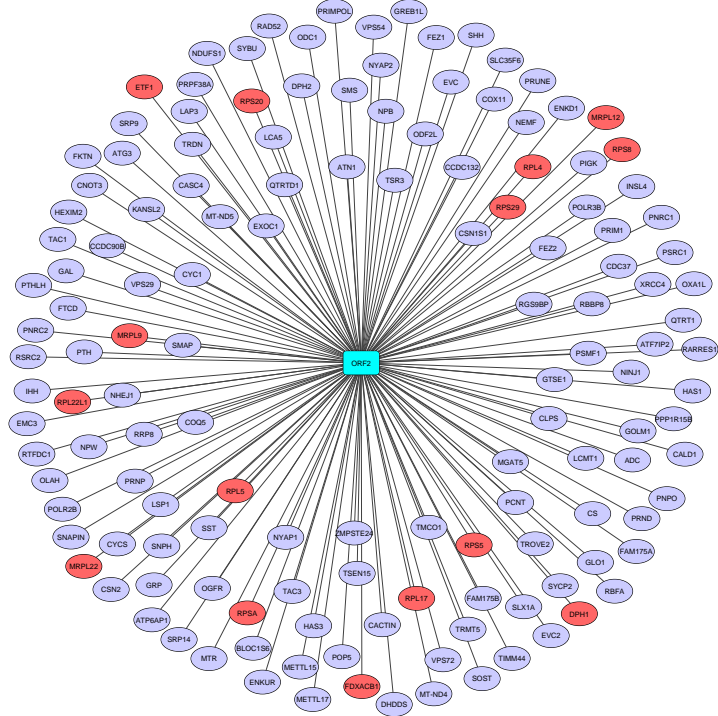

Supplement: Figure S5 — A network of HEORF2 (Genotype 1)-human protein interactions predicted by our proposed method. The network visualized by Cytoscape 3.0.2 [35]. The HEORF2 (Genotype 1) protein is representing by cyan node. The significant gene ontology enriched human proteins are representing by salmon node whereas other human proteins are representing by slate grey node. (PDF) [file pone.0112034.s005.pdf]

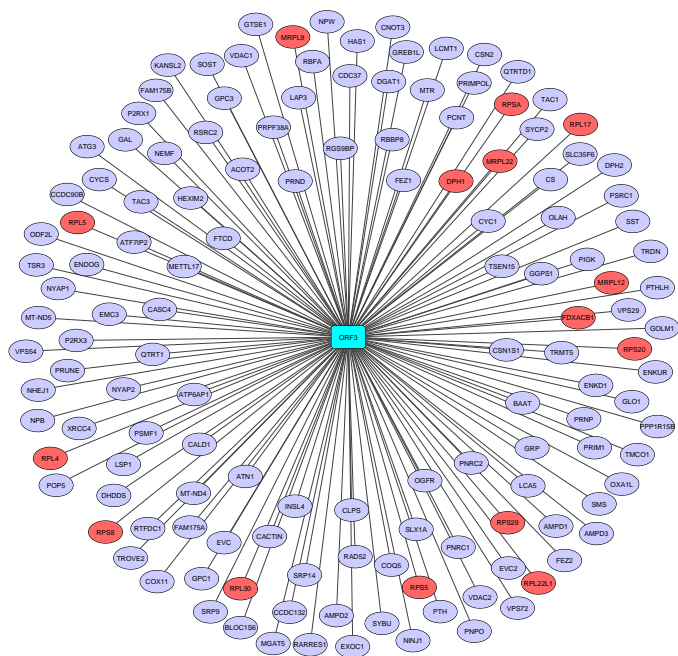

Supplement: Figure S6 — A network of HEORF3 (Genotype 1)-human protein interactions predicted by our proposed method. The network visualized by Cytoscape 3.0.2 [35]. The HEORF3 (Genotype 1) protein is representing by cyan node. The significant gene ontology enriched human proteins are representing by salmon node whereas other human proteins are representing by slate grey node. (PDF) [file pone.0112034.s006.pdf]

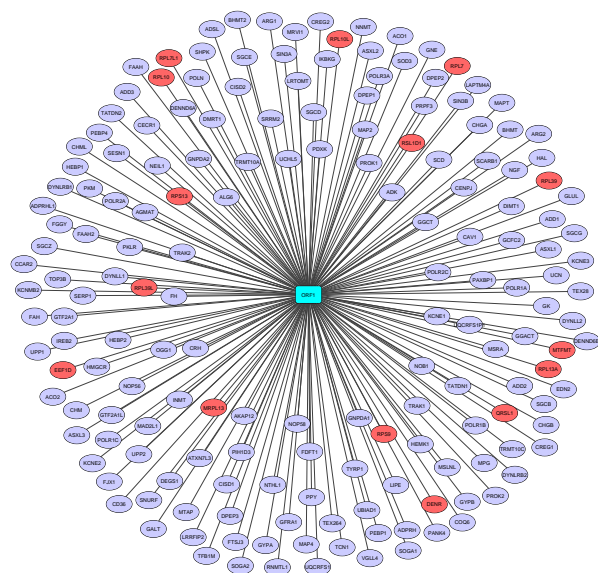

Supplement: Figure S7 — A network of HEORF1 (Genotype 4)-human protein interactions predicted by our proposed method. The network visualized by Cytoscape 3.0.2 [35]. The HEORF1 (Genotype 4) protein is representing by cyan node. The significant gene ontology enriched human proteins are representing by salmon node whereas other human proteins are representing by slate grey node. (PDF) [file pone.0112034.s007.pdf]

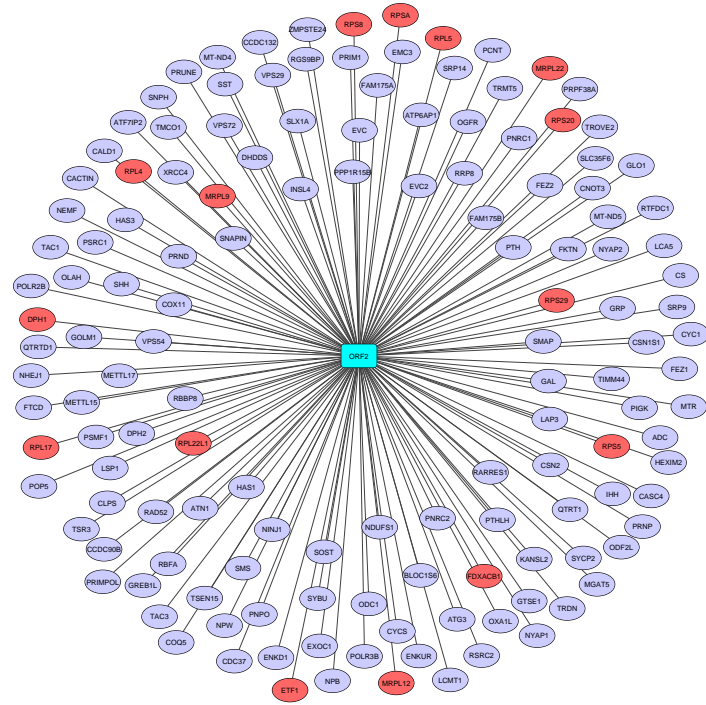

Supplement: Figure S8 — A network of HEORF2 (Genotype 4)-human protein interactions predicted by our proposed method. The network visualized by Cytoscape 3.0.2 [35]. The HEORF2 (Genotype 4) protein is representing by cyan node. The significant gene ontology enriched human proteins are representing by salmon node whereas other human proteins are representing by slate grey node. (PDF) [file pone.0112034.s008.pdf]

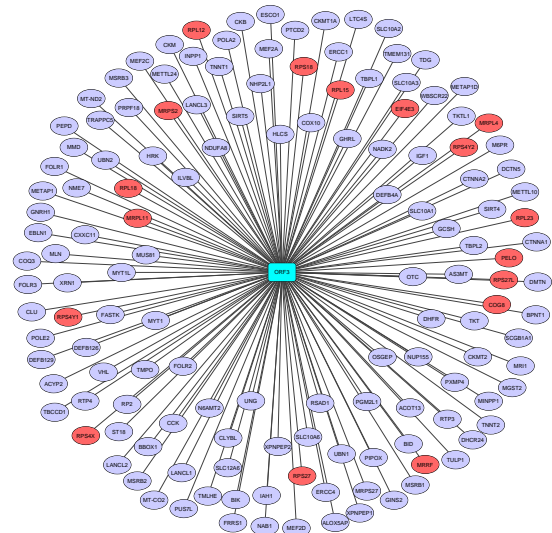

Supplement: Figure S9 — A network of HEORF2 (Genotype 4)-human protein interactions predicted by our proposed method. The network visualized by Cytoscape 3.0.2 [35]. The HEORF2 (Genotype 4) protein is representing by cyan node. The significant gene ontology enriched human proteins are representing by salmon node whereas other human proteins are representing by slate grey node. (PDF) [file pone.0112034.s009.pdf]
